# Whole-genome sequencing reveals de-novo mutations associated with nonsyndromic cleft lip/palate

**DOI:** 10.1038/s41598-022-15885-1

**Published:** 2022-07-11

**Authors:** Waheed Awotoye, Peter A. Mossey, Jacqueline B. Hetmanski, Lord J. J. Gowans, Mekonen A. Eshete, Wasiu L. Adeyemo, Azeez Alade, Erliang Zeng, Olawale Adamson, Thirona Naicker, Deepti Anand, Chinyere Adeleke, Tamara Busch, Mary Li, Aline Petrin, Babatunde S. Aregbesola, Ramat O. Braimah, Fadekemi O. Oginni, Ayodeji O. Oladele, Abimbola Oladayo, Sami Kayali, Joy Olotu, Mohaned Hassan, John Pape, Peter Donkor, Fareed K. N. Arthur, Solomon Obiri-Yeboah, Daniel K. Sabbah, Pius Agbenorku, Gyikua Plange-Rhule, Alexander Acheampong Oti, Rose A. Gogal, Terri H. Beaty, Margaret Taub, Mary L. Marazita, Michael J. Schnieders, Salil A. Lachke, Adebowale A. Adeyemo, Jeffrey C. Murray, Azeez Butali

**Affiliations:** 1 Iowa Institute for Oral Health Research, University of Iowa, Iowa City, IA USA; 2 Department of Oral Pathology, Radiology and Medicine, College of Dentistry, University of Iowa, Iowa City, IA USA; 3 Department of Orthodontics, University of Dundee, Dundee, UK; 4 Department of Epidemiology, Johns Hopkins Bloomberg School of Public Health, Baltimore, MD USA; 5 Department of Biochemistry and Biotechnology, Kwame Nkrumah University of Science and Technology, Kumasi, Ghana; 6 Surgical Department, School Medicine, Addis Ababa University, Addis Ababa, Ethiopia; 7 Department of Oral and Maxillofacial Surgery, University of Lagos, Lagos, Nigeria; 8 Department of Epidemiology, College of Public Health, University of Iowa, Iowa City, IA USA; 9 Division of Biostatistics and Computational Biology, College of Dentistry, University of Iowa, Iowa City, IA USA; 10 Department of Pediatrics, University of KwaZulu-Natal, Durban, South Africa; 11 Department of Biological Sciences, University of Delaware, Newark, USA; 12 Department of Orthodontics, University of Iowa, Iowa City, IA USA; 13 Department of Oral and Maxillofacial Surgery, Obafemi Awolowo University, Ile-Ife, Osun, A234 Nigeria; 14 Department of Anatomy, University of Port Harcourt, Choba, Nigeria; 15 Department of Surgery, School of Medicine and Dentistry, Kwame Nkrumah University of Science and Technology, Kumasi, Ghana; 16 Department of Maxillofacial Sciences, School of Medicine and Dentistry, Kwame Nkrumah University of Science and Technology, Kumasi, Ghana; 17 Department of Child Oral Health and Orthodontics, School of Medicine and Dentistry, Kwame Nkrumah University of Science and Technology, Kumasi, Ghana; 18 Department of Child Health, School of Medicine and Dentistry, Kwame Nkrumah University of Science and Technology, Kumasi, Ghana; 19 Center for Biocatalysis and Bioprocessing (CBB), University of Iowa, Iowa City, USA; 20 Center for Craniofacial and Dental Genetics, Department of Oral and Craniofacial Sciences, School of Dental Medicine, and Department of Human Genetics, Graduate School of Public Health, University of Pittsburgh, Pittsburgh, PA USA; 21 Center for Bioinformatics and Computational Biology, University of Delaware, Newark, USA; 22 National Human Genomic Research Institute, Bethesda, MD USA; 23 Department of Pediatrics, University of Iowa, Iowa City, IA USA

**Keywords:** Computational biology and bioinformatics, Developmental biology, Genetics, Medical research, Molecular medicine, Risk factors

## Abstract

The majority (85%) of nonsyndromic cleft lip with or without cleft palate (nsCL/P) cases occur sporadically, suggesting a role for de novo mutations (DNMs) in the etiology of nsCL/P. To identify high impact protein-altering DNMs that contribute to the risk of nsCL/P, we conducted whole-genome sequencing (WGS) analyses in 130 African case-parent trios (affected probands and unaffected parents). We identified 162 high confidence protein-altering DNMs some of which are based on available evidence, contribute to the risk of nsCL/P. These include novel protein-truncating DNMs in the *ACTL6A, ARHGAP10, MINK1, TMEM5* and *TTN* genes; as well as missense variants in *ACAN, DHRS3, DLX6, EPHB2, FKBP10, KMT2D, RECQL4, SEMA3C, SEMA4D, SHH, TP63,* and *TULP4*. Many of these protein-altering DNMs were predicted to be pathogenic. Analysis using mouse transcriptomics data showed that some of these genes are expressed during the development of primary and secondary palate. Gene-set enrichment analysis of the protein-altering DNMs identified palatal development and neural crest migration among the few processes that were significantly enriched. These processes are directly involved in the etiopathogenesis of clefting. The analysis of the coding sequence in the WGS data provides more evidence of the opportunity for novel findings in the African genome.

## Introduction

Nonsyndromic clefts of the lip with or without cleft palate (nsCL/P) represent one of the most common types of birth defect in humans and the most common of the craniofacial region^1^. These are developmental malformation resulting from the failure of the well-coordinated complete fusion of the facial prominences during embryogenesis^2^. This birth defect is one of the sub-types of nonsyndromic orofacial clefts (nsOFCs) which accounts for 70% of the orofacial clefts (OFCs). Other sub-types of nsOFCs include nonsyndromic cleft palate only (nsCPO). The combined global prevalence of OFCs is reported to be 1 in 700 livebirths^3^.

Associated impairments due to these malformations include feeding problems, speech defects, malocclusion, and esthetics problems. Studies have reported a significant increase in overall mortality in people with nsCL/P^4^. Families of affected individuals are often stigmatized and have reported huge burdens on their financial, psychological and social well-being^5^. Effective management involves a team of specialists who conduct surgical repair of the defect and manage challenges in dental, speech and psychology^6,7^. Due to the need for multi-disciplinary expertise over the life course from birth to adulthood, the cost of management and the negative impact on oral health-related quality of life, nsCL/P poses a huge public health burden.

Genetic and environmental factors have been shown to contribute to the risk of nsCL/P at the population level. The role of genetics in the etiology of clefts have been well-reported with an estimated heritability rate between 50 and 80%^8^. Although all cleft cases tend to show familial tendency but only about 40% have been causally linked to genetic risk factors^9^. Familial aggregation of orofacial clefts indicates the contribution of genetics to the risk of these defects. A study found that first degree relatives of families with CL/P have 32 times higher risk of recurrence compared to those without positive family history^10^. This study also reported that the relative risk of CP recurrence among first degree relative is 56 times higher than that of the population-based control^10^. As the degree of segregation increases, the relative risk of clefts in affected families continues to reduce^10^. This is evident from the relative risks in third degree relatives not significantly different from population-based control^10^. Additionally, twin studies reported a concordance rate of clefts in monozygotic twins (40–60%) to be higher than that reported in dizygotic twins (3–5%)^11^. Other studies have reported that a nsCL/P affected parent has a 3.2% chance of having an affected offspring^10^. Following the birth of an affected child to such parent, the risk of the defect in another birth increases to 15.8%^10^. However, unaffected parents with an affected child have a 4.4% risk of having another child born with nsCL/P^10^. Putting these together, a strong genetic component in the etiopathogenesis of clefts becomes discernable.

Despite extensive genome-wide association studies (GWAS) which have identified about 60 common risk loci, ~  75% of the estimated heritability of liability to nsCL/P remains unexplained^12–22^. The contribution of rare coding variants has also been investigated to identify some of this missing heritability^23–25^. However, a significant knowledge gap still remains in our understanding of the genetics of nsCL/P. nsCL/P can either be sporadic or familial. Majority (80%) of the cases are sporadic, thus, suggesting a role for de novo mutations (DNMs)^26^. A few studies have examined the role of these DNMs in the etiology of nsCL/P through targeted sequencing analysis of candidate genes in affected families^27,28^. With the advent of next-generation sequencing, discovery of DNMs that may contribute to the risk of nsCL/P has yielded more positive results. The first large scale whole-genome sequencing (WGS) study reported an enrichment of DNMs in multi-ethnic nsCL/P case-parent trios of European, Colombian, or Taiwanese ancestry^29^. However, the role of these DNMs is yet to be studied on the African continent which has populations with the most genetic diversity and provides opportunity for novel findings^30^.

Using the WGS data from nsCL/P African case-parent trios generated as part of the Gabriella Miller Kids First (GMKF) Pediatric Research Consortium, here we investigated the role of high impact DNMs and here we identify some that could increase the risk of nsCL/P.

## Results

### Samples and variants filtration

**Figure 1:**
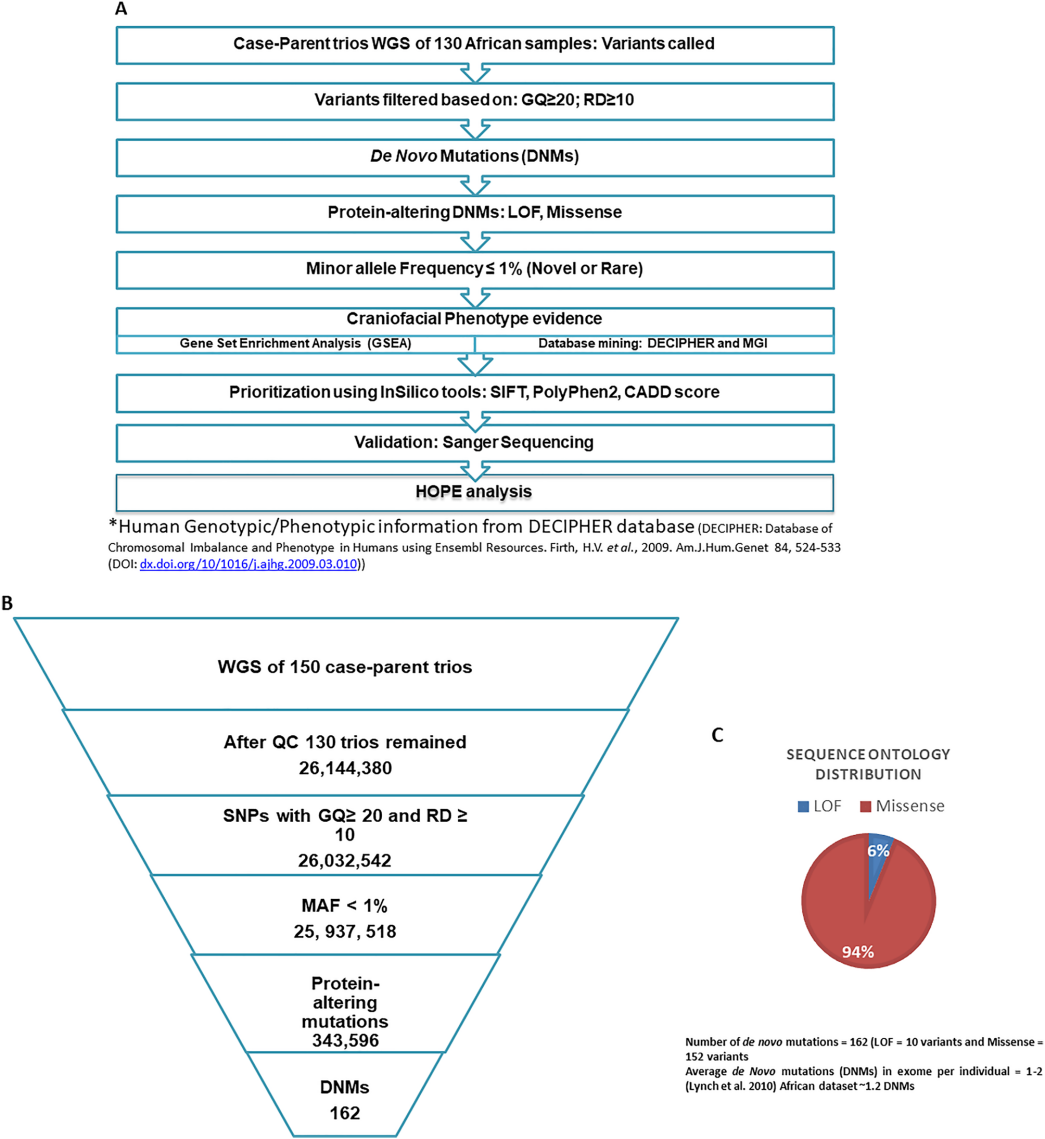
Case-parent trios’ definition, cleft sub-types, and Data filtration pipeline. (**A**) Data filtration pipeline used to identify the high confidence de novo mutations (DNMs) that contribute to the risk of nsCL/P. (**B**) Details of the number of variants from each data filtering steps which resulted in 162 DNMs in protein-coding genes. (**C**) Pie chart showing the distribution of the effects of the de novo Variants. Majority of the DNMs (94%) cause amino acid changes which alters the protein structures and functions while 6% cause loss of function mutation in the protein-coding genes.

Following our deep phenotyping and samples recruitment through the African Craniofacial Anomalies Network (AfriCRAN)^14^, 150 case-parent trios (i.e., each trio consists of an affected child and unaffected parents as depicted in Fig. 1A) were selected for whole-genome sequencing at the Broad Institute. The ethnicity was determined at the point of recruitment and during the quality control. At the point of recruitment, case-parent trios were included provided their parent and grandparents were of African descent. Additionally, our quality control confirmed that these cohorts’ principal components of ancestry (PCA) clustered with the HapMap sample PCs from west African countries. These samples were part of the cohorts used in the first African cleft GWAS which has been published^14^.

The quality control (QC) process checked for completeness of the sequenced genomes, Mendelian errors and relatedness; this resulted in dropping 20 trios. The variants from the remaining 130 case-parent trios were filtered for high quality by ensuring that each had a genotype quality of at least 20 (GQ ≥ 20) and read depth of at least 10 (RD ≥ 10). These high-quality variants were further filtered to identify those predicted to have a high impact on the gene product and present in the case but not the parents, i.e., a de novo mutation (DNM) (MAF < 1%; protein-truncating and missense consequences). Details of the number of variants from each data filtering steps are shown in Fig. 1B. This resulted in the identification of 162 protein-altering DNMs which averages 1.2 DNMs within the coding region per case (Fig. 1C). All the protein-altering DNMs were sanger validated.

### Novel protein-altering DNMs contribute to the risk of nsCL/P

We found 162 protein-altering DNMs (Supplementary Table 1) 17 of them in genes recognized to play roles in craniofacial development and potentially contribute to the risk of nsCL/P (Table 1). Copy number variations (CNVs) involving these genes indicate that these genes could well be involved in craniofacial morphogenesis (Table 1). Mouse knockouts of *Acan,Dhrs3, Kmt2d, Recql4, Shh* and *Tp63* showed orofacial cleft phenotypes. The remaining genes: *ACTL6A, ARHGAP10, FKBP10, MINK1, TMEM5, TTN* and *TULP4* lack mouse knockout models to support their involvement in OFCs but other craniofacial dysmorphologies have been reported. Knockout of *Actl6a* in mice was reported to be embryonically lethal as the mice did not survive beyond developmental stage E6.5^31^.

**Table 1:**
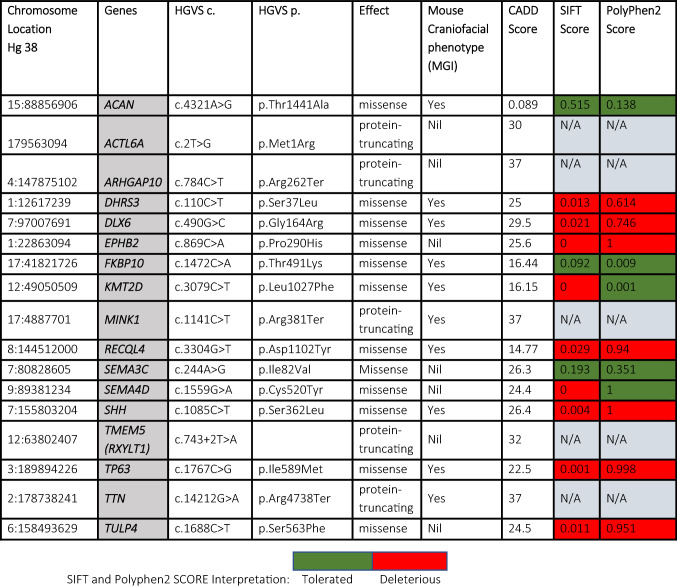
List of novel variants which have evidence suggestive of involvement in craniofacial development and role in development of nsCL/P.

These evidences are based on the phenotype in humans with CNVs involving these genes as well as SNVs which are reported in the DECIPHER database (https://www.deciphergenomics.org/).

*Hg38: Human genome build 38 (GRCh38).

*Nil: No craniofacial phenotype in mouse studies; N/A: Insilico scores not available for protein-truncating mutations.

The DNM in *TTN* was found in the exon 49 which changed the codon for the amino acid Arginine to a stop codon at position 4738. The consequence of this premature stop codon is a truncation in the polypeptide chain and hence result in a loss of function of the gene product. Similar mutation consequences were found in the *ARHGAP10* and *MINK1* genes. However, the protein-truncating mutations in *ACTL6A* and *TMEM5* are due to an initiator codon and splice donor variants, respectively. The missense mutation in *DHRS3* changes a Serine amino acid to leucine at position 37. This position falls in the *DHRS3* catalytic domain which is critical for its enzymatic function in vitamin A metabolism. The mutation in *TP63* lies in the highly conserved sterile alpha motif (SAM) domain which is critical for protein–protein interaction of the molecule. Interestingly, the damaging protein-altering DNM discovered in *SHH* gene (p.Ser362Leu) in this study has been previously reported as the cause of a syndromic cleft(OMIM #142945)^32^. Following this discovery, a detailed review of medical records of the case carrying this DNM was done but we found no evidence of any other structural birth defect. This suggests that this mutation may contribute to the risk of syndromic as well as nonsyndromic clefts^33^.

### Bioinformatics analysis showed pathogenicity of identified DNMs

The pathogenicity of these DNMs was predicted using in-silico tools to investigate the effect of the DNMs on gene expression, protein structures and functions. We used Combined Annotated Dependent Depletion (CADD) tool to ascertain the deleteriousness of the nucleotide changes that caused the DNMs. Additionally, we used Sorting Intolerant From Tolerant (SIFT) and Polymorphism Phenotyping (PolyPhen2) tools to predict how damaging the amino acid changes (resulting from the DNMs) are to the protein; and finally used HOPE to identify the effect of the amino acid changes on the protein structure and function. DNMs in the *ACTL6A, ARHGAP10, MINK1, TMEM5* and *TTN* were predicted to be among the top 0.1% most deleterious mutations in the human genome (see Table 1 for CADD scores). The DNMs in the *DHRS3, SHH, TP63* and *TULP4* genes were predicted to be among the top 1% most deleterious mutations in the human genome (see Table 1 for CADD scores) while the other genes (DNMs in *ACAN, FKBP10, KMT2D* and *RECQL4*) are among the top 10% deleterious mutations (Table 1). Among the missense DNMs, only variants in *ACAN* and *FKBP10* were predicted by both SIFT and PolyPhen2 to be well tolerated and are benign. Other missense DNMs were predicted by at least one of the two in-silico tools to be deleterious or damaging (Table 1).

Our analysis of the effect of the amino acid changes due to these missense DNMs showed that the resulting alterations in the protein structures could impact the functions of the molecules encoded by the mutated genes. Notably, the secondary structures and the protein interactions are affected (Supplementary Fig. 1). The amino acid changes identified in DHRS3, DLX6, SEMA3C, SEMA4D, SHH and TP63 occur at highly conserved region and/or critical domains (Supplementary Fig. 1).

The SHH mutation p.Ser362Leu we discovered lies in a highly conserved region of the protein; the serine residue is the only amino acid at this location in available vertebrate orthologs going back to Zebrafish. This DNM occurs within the Hedgehog (Hh) domain located at the carboxyl terminal of the polypeptide. This region is important for protein auto processing, thus modifying of the N terminal of the protein which is critical for the protein interactions which mediates Hh signaling^34,35^.

**Figure 2:**
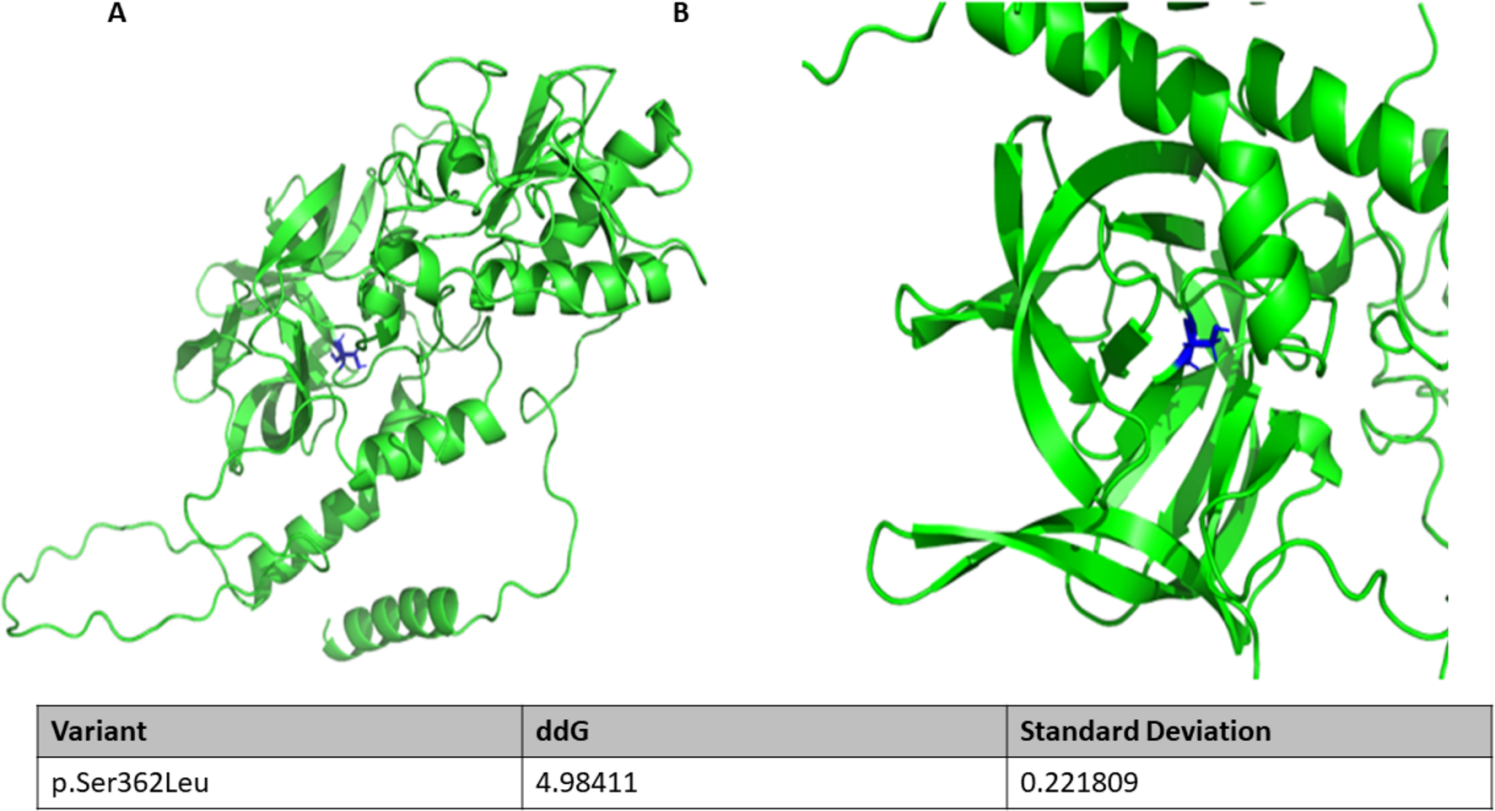
AlhpaFold predicted protein structures of the SHH (**A,B**). Highlighted in blue is the side-chain where the mutation occurred ((**A**) closed-up view in (**B**)). This is located within the Hedgehog domain that is critical for the hedgehog signaling. Table shows thermodynamic prediction of the effect of the p.Ser363Leu SHH DNM on the protein stability. The amino acid change resulted in a change in the folding free energy by 4.984 kcal/mol (± 0.221). This change is predicted to be disease causing.

Using the updated neural network-based model called AlphaFold2, we computationally predicted the 3-dimensional structure of the SHH variants^36,37^. We then estimated the folding free energy (∆G) change through thermodynamic analysis. First, this calculated the free-energy of the unfolded SHH variant then that of the folded variants. We then estimated the difference between these free-energies and used the value to predict the protein stability^36,37^. This analysis estimated the value of folding free energy change (∆∆G) associated with this p.Ser362Leu variant to be 4.984 kcal/mol with a standard deviation of 0.221. The folding free energy change measures the protein stability resulting from the amino acid change due to this mutation. The mutation causes a positive folding free energy change (∆∆G) which indicates that it is destabilizing the protein structure^37^ (Fig. 2).

### Palate development and neural crest migration biological processes are significantly disrupted by DNMs

**Figure 3:**
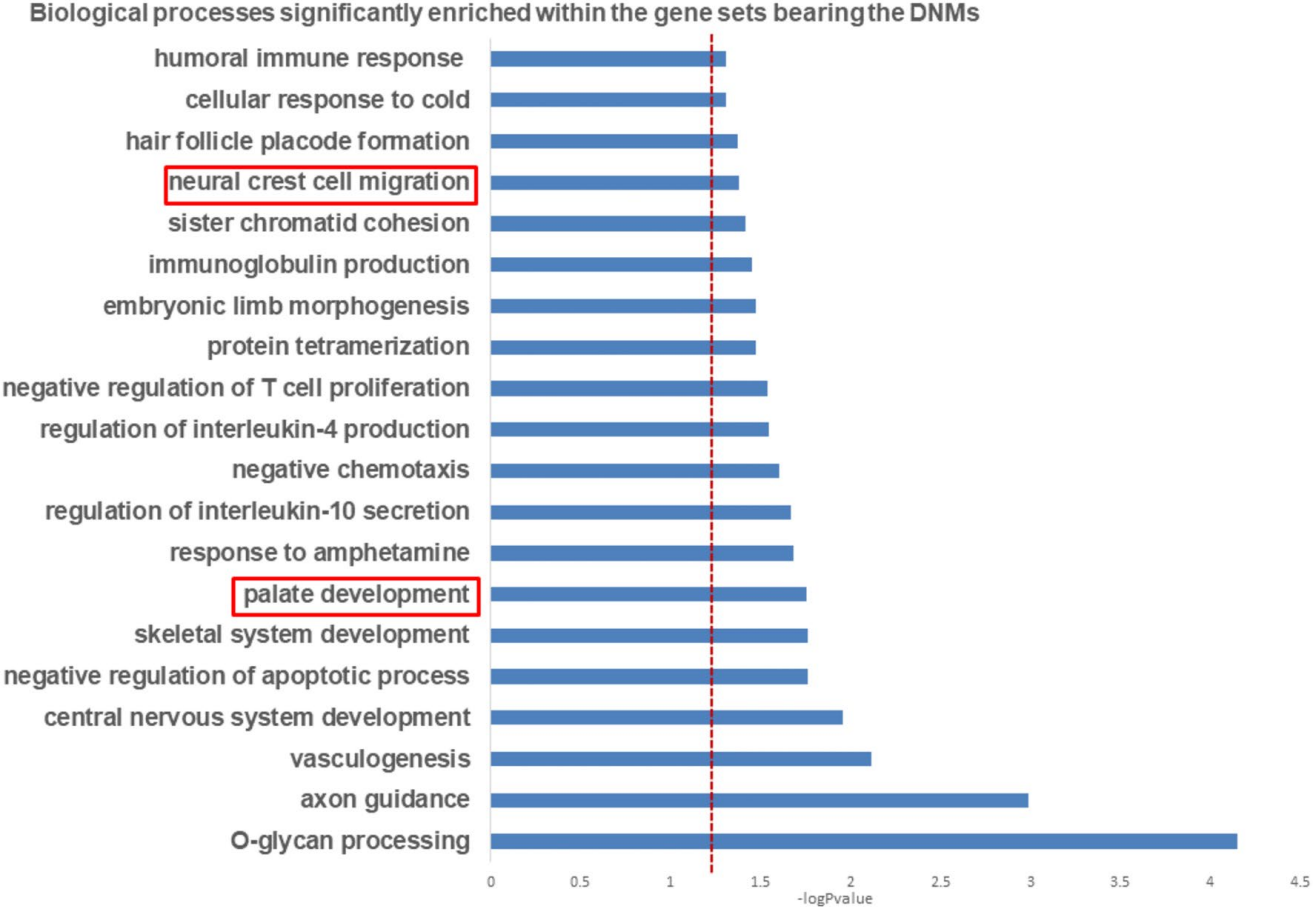
Graph showing significantly enriched BP from GSEA. The palate development and neural crest migration are among the processes significantly enriched (p < 0.05).

Our gene set enrichment analysis (GSEA) identified significantly enriched processes (p < 0.05) involved in normal development of the lip and palate (Fig. 3). Among these biological processes showing at least nominal significant enrichment, we identified *palate development and neural crest migration* (p values are 0.02 and 0.04; respectively). These biological processes have been causally linked to the etiopathogenesis of OFCs as disruption could manifest as craniofacial dysmorphology such as OFC.

Among our list of prioritized genes, the genes known to play significant roles in the development of the palate are *DHRS3*, *DLX6, EPHB2* and *SHH,* while those contributing to neural crest migration include *SEMA3C, SEMA4D* and *SHH*. This GSEA analysis identified *DLX6, EPHB2, SEMA3C* and *SEMA4D* as potential cleft candidate genes.

### SysFACE analysis informs on gene expression in facial tissue development

**Figure 4:**
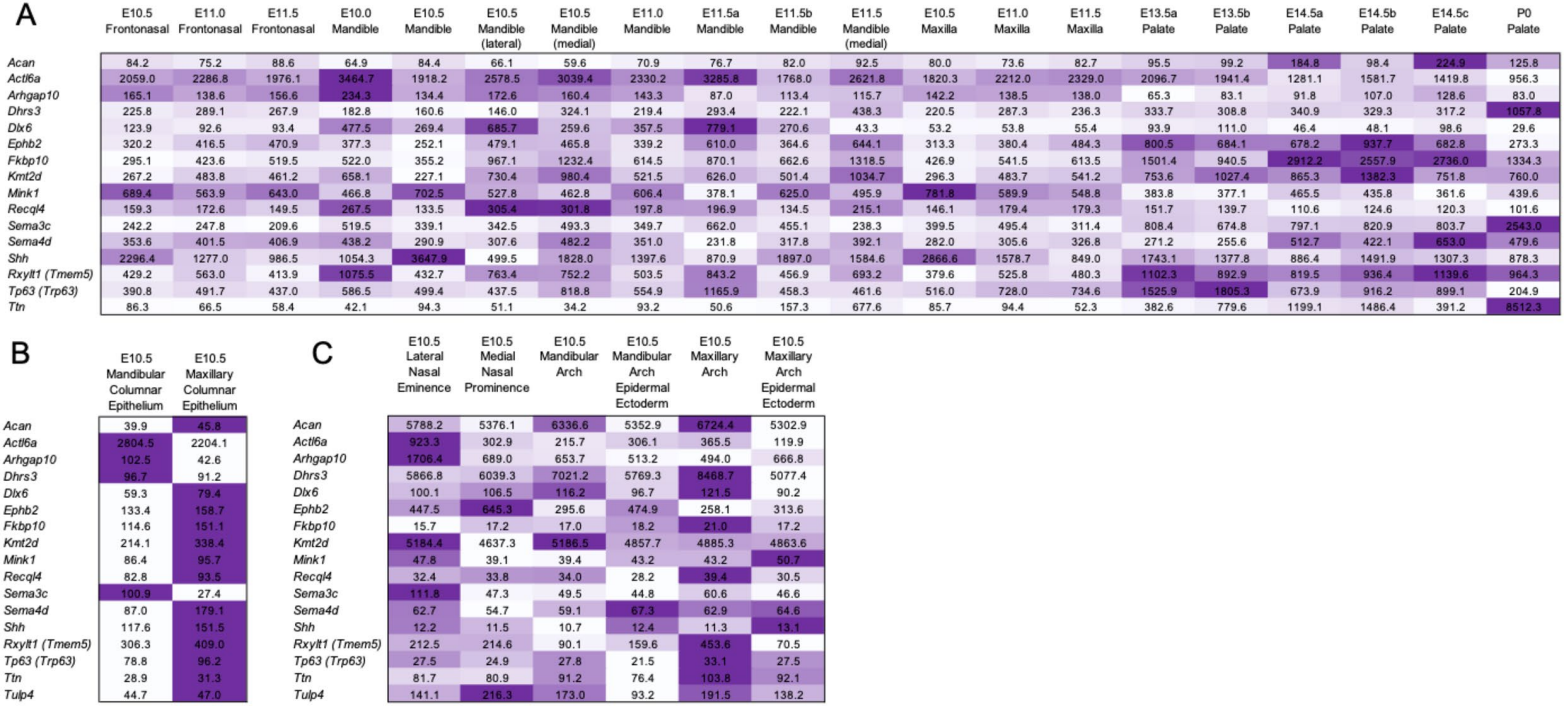
SysFACE-based expression analysis of candidate genes in mouse facial development**.** Expression of candidate genes based on analysis using (**A**) GSE7759 microarray data generated on the Affymetrix Mouse Genome 430 2.0 Array platform, (**B**) FaceBase microarray data generated on the Affymetrix Mouse Gene 1.0 ST Array platform, and (**C**) GSE55965 microarray data generated on the Affymetrix Mouse Gene 1.0 ST Array platform. Heat-map denotes row-wise comparative expression of individual genes in different tissues at Embryonic (E) and/or postnatal (P) stages. Intensity of the color in the heat-map is representative of candidate gene expression and the average fluorescence signal intensity is shown.

Using Systems tool for craniofacial expression-based gene discovery (SysFACE), we found that several genes with DNMs exhibit expression in mouse facial tissue development and are likely to contribute to the development of lip and palate (Fig. 4, Supplementary Fig. 2). The expression profiles showed that except for that of *TULP4* (whose ortholog was not detected in mouse), all the genes in Table 1 were found to be highly expressed in several facial tissues (Fig. 4A). Furthermore, majority of these candidates exhibit elevated expression in the E10.5 maxillary columnar epithelium compared to the E10.5 mandibular columnar epithelium (Fig. 4B). Similarly, majority of these candidates showed elevated expression in E10.5 maxillary arch compared to E10.5 mandibular arch (Fig. 4C). Several genes showed highest expression in the palate tissue (Fig. 4A). Moreover, SysFACE identified several other candidate genes that were expressed in mouse facial development (Supplementary Fig. 2).

## Discussion

To our knowledge, this case-parent trios’ analysis of the coding sequence in the genome to identify high impact protein-altering DNMs that could contribute to the risk of nsCL/P is a first of such analysis in an African population. Our approach utilized a trio-based study design and analysis to identify potentially pathogenic variants that can explain sporadic cases of nsCL/P. Our analysis also incorporated data from publicly available databases to identify those genes that could play a role in craniofacial development in animal models. We identified several high impact protein-truncating and missense DNMs that appear to contribute to the risk of nsCL/P. Additionally, we used thermodynamic analysis to investigate the effect of the amino acid change on the protein stability.

The protein-truncating DNMs were found in *ACTL6A, ARHGAP10, MINK1, TMEM5* and *TTN* genes, all of which are loci with substantial annotation, functional and/or animal model data supporting their roles in orofacial clefts. *ACTL6A* encodes an actin-related protein involved in chromatin remodeling and knockout mice do not survive beyond E6.5^31^. *ARHGAP10* is a member of the Rho GTPase family that is important in cell adhesion, migration and proliferation. This rho GTPase regulates WNT signaling by reducing the expression of *CTNNB1*^38^. The dysregulation of WNT signaling has been well reported in the etiology of cleft^39^. *MINK1* encodes misshapen-like kinase 1 which functions in cell–cell adhesion and migration. Mutant mice showed an abnormal tooth morphology indicating this gene plays some role in craniofacial development^31^. *TMEM5* encodes a transmembrane protein and mutations have been implicated in neural tube defects with some affected individuals presenting with clefts^40^, further GWAS found association between common SNPs in non-coding regions near this gene family and CL/P^12^. Titin protein (encoded by *TTN*) is the largest protein molecule that plays a role in the development of the striated muscles. Genetic mutations in this gene cause congenital titinopathy: a birth defect characterized by myopathies (with cardiomyopathy)^41^. Cleft palate has also been reported in some individuals with this birth defect^42^. Mutant mice showed an increased apoptosis of cells in the frontonasal process, an important tissue that contribute to the development of the lip and palate and could easily contribute to development of OFCs^43^. Our expression analysis using the SysFACE tool provides additional evidence suggesting some role for the DNMs in *ACTL6A, ARHGAP10, MINK1* and *TMEM5* in the etiopathogenesis of nsCL/P. However, experimental evidence in model animals would confirm the role of these genes in the development of the lip and palate; and may shed light on these DNMs in the etiology of cleft.

Our analysis also found several missense DNMs in genes recognized as contributing to the risk of nsCL/P. We found a damaging mutation in *DHRS3,* which encodes an enzyme important in the metabolism of retinol. The DNM we identified is in the catalytic domain which is critical for enzymatic function. Mouse knockout models for this gene resulted in the cleft palate phenotype seen at E14.5^44^. Here, we report a damaging missense DNM in *TULP4* which encodes tubby-related protein 4 which functions in post-translation modification. This gene has only been reported in association studies where it was found to be associated with orofacial cleft in Filipinos and in Africans (Ethiopia, Ghana and Nigeria)^45,46^. We also identified damaging missense DNMs in cleft candidate genes *SHH* and *TP63.* The damaging mutation in *TP63* lies within a highly conserved sterile alpha motif (SAM) domain. Damaging mutations within this SAM domain have been reported to cause clefts^47,48^ and genome-wide approaches found significant association between nsCL/P and common SNPs within *TP63*^13,49,50^.

Other genes which have damaging DNMs identified in our study include *KMT2D* and *RECQL4. KMT2D* encodes a methyl transferase which functions in transcription activation. It has been reported to be associated with Kabuki syndrome in both humans and mice^51^. Protein-truncating *Kmt2d* in the neural crest cells result in a fully penetrant cleft palate phenotype^52^. *RECQL4* encodes a helicase which plays a role during DNA replication. Mutations in this gene have been associated with autosomal recessive Rapadilino syndrome (OMIM #266280) which in addition to limbs, joints and knee anomalies, and affected individuals may present with cleft palate^53,54^. Mutant mice recapitulated most of these phenotypes (including cleft palate)^55^. Although the DNMs reported in *FKBP10* and *ACAN* that encode a binding protein and an extracellular matrix protein (aggrecan) are predicted to be benign, studies have suggested they play roles in craniofacial development^56^. In-vitro studies and knockout experiments in mice provide evidence of the role of aggrecan in the etiology of cleft^57,58^.

Gene-set enrichment analysis identified other genes on our list whose DNMs may also contribute to the risk of nsCL/P. These genes are involved in biological processes; palate development and neural crest migration which have been directly linked with the etiopathogenesis of orofacial clefts^59^. Although the distal-less homeobox 6 (*Dlx6)* mouse knockouts show a number of craniofacial defects, cleft phenotypes have not been reported. The ephrin type B receptor 2 (*Ephb2*) is a member of the ephrin cell membrane receptors which bind to each other and initiate the Eph and ephrin signaling. The forward signaling of the bidirectional (forward-Eph and reverse-ephrin)signaling pathways is critical for normal palatogenesis^60,61^. The truncation of Ephb2 in an Ephb3 null (EphB2^lacZ/lacZ^/EphB3^−/−^) mice inhibited the proliferation of the palatal mesenchyme which resulted in cleft palate^60^*.* The sema domain (semaphorin) 3C and 4D (*SEMA3C* and *SEMA4D)* mouse knockout do not display craniofacial defects but are expressed in the 1st branchial arches which contribute indirectly to the development of the lip and palate^31^. Additionally, homozygous *Sema3c* knockout is perinatally lethal. These genes are highly expressed in the embryonic tissues critical to normal development of the lip and palate. With the aid of this SysFACE tool, we also identified novel genes contributing to the development of the lip and palate. We deduced DNMs in these genes may contribute to the risk of cleft in humans. Among other genes identified with the SysFACE tool, *MMP9* has been reported to be a key extra cellular matrix remodeling protein that could play a role in lip and palate development^62^. *Junb, Jup* and *Dnajc3* are among the genes highly expressed during the 3-way fusion that forms the lamdoidal junction in the developing face^63^. This 3-way fusion comprises fusion of the medial nasal process (MNP), lateral nasal process (LNP) and the maxillary process (MxP): MNP and LNP; MNP and MxP; LNP and MxP and is critical in the development of the lip and palate^63^.

In conclusion, our analysis of the WGS in African nsCL/P case-parent trios led to the discovery of novel pathogenic genetic mutations likely to contribute to the risk of OFC. The findings of nsCL/P-risk protein-altering DNMs in some of these genes for the first time expands our knowledge of the genetic architecture of sporadic nsCL/P and provides further evidence to support the role of de novo mutations in the risk of the most common craniofacial birth defect.

## Materials and methods

### Study samples

All the individuals recruited were of African ancestry from the 2 participating countries (Ghana and Nigeria). As per the established AfriCRAN protocol developed by Butali and colleagues, recruitments of infants with OFC and their parents were done during the evaluation of the affected child for surgical repair of their clefts. We recruited children affected with nsCL/P and the unaffected parents (father and mother) i.e., case-parent trios for this genetic study. In some situations, we recruited just mother and affected child i.e., dyads and in rare occasions, we recruited other family members like siblings and grand-parents. For the current study, only case-parent trios were included. Before a trio was recruited, the parents and grandparents must be ascertained to be of African ancestry and have reported no family history of any major birth defect. Following the study design, ethical approvals were obtained at the local institution review boards (IRBs) at the participating sites: Lagos University Teaching Hospital (ADM/DCST/HREC/VOL.XV/321), Obafemi Awolowo University Teaching Hospital (ERC/2011/12/01), Kwame Nkrumah University of Science and Technology (CHRPE/RC/018/130) and University of Iowa (IRB ID #: 201101720). The methods used in the recruitment at the different centers were carried out in accordance with statutory guidelines and regulations. Informed consent was obtained from all subjects included in this study.

A case-parent trio was recruited following deep phenotyping of the type of cleft and ruling out other congenital anomalies. This ensured the case (affected child) had a nonsyndromic cleft phenotype while the parents were unaffected. A standardized phenotyping protocol was used by the surgeons during the physical examination, taking clinical photographs and detailing the cleft phenotypes in a clinical database as reported in our previously published works^14,64^. Echocardiography was used to rule out congenital cardiac defects. For each trio, the cleft status describing the type of cleft was recorded. Table 2 shows the number of trios, site of recruitment and their cleft status. The distribution of the cases based on cleft types is as shown in Table 2.

**Table 2:** Distribution of the case-parent trios based on the country of origin and cleft status.

Country	Cleft status		Total
	Cleft lip	Cleft lip and palateCleft lip and palate	
Ghana	40	64	104
Nigeria	11	15	26
	51	79	130

Majority (79) of the trio have CLP. Each trio consisted of a nsCL/P affected child and unaffected parents.

SIFT and Polyphen2 SCORE Interpretation: Tolerated Deleterious.

Saliva samples were collected from the parents and the affected child using the Oragene saliva tool kits. Each case-parent trio was assigned a unique identifier number and their epidemiological and clinical information were remotely uploaded into a secure REDCap database. Following de-identification, the saliva samples were shipped to the Butali laboratory at the University of Iowa for processing.

### DNA extraction and XY genotyping

Saliva samples received from the recruitment centers were labeled with their unique identifier (UNID) number. The DNA was isolated from the saliva samples using the Oragene DNA extraction protocol. Extracted DNA samples were quantified using Qubit (http://www.invitrogen.com/site/us/en/home/brands/Product-Brand/Qubit.html; ThermoFisher Scientific, Grand Island, NY). Stocks and working aliquots of each DNA samples were made for future testing.

We confirmed the reported sex using the TaqMan XY genotyping. Confirmation of the sex is an inhouse quality control (QC) used in the Butali laboratory. Working aliquots (25 μl) passing QC with DNA concentration ≥ 250 ng were shipped to the Broad Institute for whole genome sequencing supported by the Gabriella Miller Kids First program.

### Whole-genome sequencing and variant calling

Our nsCL/P case-parent trios’ DNA samples were part of the cohorts sequenced under the Gabriella-Miller Kids First (GMKF) Pediatric Research Consortium (https://kidsfirstdrc.org/). This consortium was established and funded to address the knowledge gaps in the understanding role of the genetics in the etiology of structural birth defects and pediatric cancers. The WGS was conducted at the Broad Institute with entire genome sequenced an average of 30 times (30 × WGS). The binary alignment map (BAM) and sequence alignment map (SAM) files were obtained after the sequence data were aligned to the Human genome assembly GRCh38 (hg38). Alternate alleles (i.e., variants from the reference genome), were called when present using the GenomeAnalysisToolKit (GATK) pipelines at the Broad Institute (https://software.broadinstitute.org/gatk/best-practices/workflow). Briefly, these variants include single nucleotide variants (SNVs) and Insertions or deletions (Indels), were called using the HaplotypeCaller in GVCF mode and GenotypeGVCFs for single-sample variant calling and the multiple-sample joint variant calling respectively. Variants were stored in a variant call format (VCF) file which was used for further analyses.

### Quality control

The quality control of the case-parent nsCL/P African trios WGS data was done using PLINK v.1.9. Each individual in a case-parent trio were evaluated on variety of quality metrics. Individuals with missingness > 10%, inconsistency between the sex reported and the average homozygosity of X-chromosome or Hardy–Weinberg Equilibrium (HWE) < 1E−06 were excluded. Also, trios showing deviation from the expected degree of relatedness between the case (offspring) and parents, or case-parent trios with Mendelian errors outside three standard deviations from the mean were excluded. Additionally, individuals with variant calls beyond 4 standard deviations from mean heterozygote/homozygote ratio were excluded.

A case-parent trio was retained only when the offspring and parent samples meet these quality control thresholds. If at least one sample in a trio does not meet these thresholds, the entire case-parent trio was excluded. After the quality control, 130 out of 150 case-parent trios were retained for downstream analyses.

### Analyses for de novo mutations (DNMs) contributing to risk of nsCL/P

Following the variant calling, we filtered for high confidence protein-altering DNMs using the data filtration pipeline in Fig. 1C. Variants were first filtered based on genotype quality (GQ) ≥ 20 and a read depth (DP) ≥ 10. The high-quality variants were then filtered for mutations present in the affected case but absent in the unaffected parents (DNMs). Potential DNMs passing these filtering steps were then examined for high impact/ protein-altering mutations. Such mutations are within the coding region of the genes and the selected consequences are protein-truncating and missense mutations creating altered gene products.

Following the identification of these coding DNMs, we filtered for those variants with minor allele frequency (MAF) ≤ 1% (0.01). We did this by comparing the identified DNMs to variants reported in the 1000 Genome database (https://www.internationalgenome.org/), Exome Variant Server database (https://evs.gs.washington.edu/EVS/) and Genome Aggregation Database (https://gnomad.broadinstitute.org/). Allelic frequencies in these public databases contains whole genome sequencing data from over 7000 African and African-American controls including individuals from Ghana and Nigeria.

We then identified those genes with DNMs with some evidence of involvement in human craniofacial development. This was achieved by mining the DECIPHER database (https://www.deciphergenomics.org/) to identify those genes with copy number variants (CNVs), indels and SNVs reported in individuals with craniofacial anomalies. We prioritized those genes recognized as associated with lip and palate anomalies or anomalies in other craniofacial structures. In a bid to identify the contribution of these DNMs to the risk of nsCL/P, we also mined the Mouse Genome Informatics (MGI) database. We focused on genes among our list with cleft phenotype in mouse knockouts.

Next, we predicted the pathogenicity of these protein-altering DNMs using the bioinformatic tools such as Sorting Intolerant From Tolerant, SIFT (http://sift.jcvi.org/)^65^. Polymorphism Phenotyping, PolyPhen2 (http://genetics.bwh.harvard.edu/pph2/)^66^ and Combined Annotation Dependent Depletion, CADD (https://cadd.gs.washington.edu/)^67^. We identified protein-altering DNMs predicted to be deleterious, damaging or among the topmost deleterious mutations in the human genome.

Furthermore, we investigated the effect of missense DNMs on the protein structure and function. We used the bioinformatic tool, Help you Protein Explained: HOPE (https://www3.cmbi.umcn.nl/hope/)^68^ to predict effects of the amino acid change on the protein structure and function. Additional computational methods were used to predict the structural effects of the DNM on one of the most reported cleft candidate gene products. Starting from the predicted protein structures generated by AlphaFold2, we locally optimized the structure to relax its backbone torsions and performed sidechain optimization (i.e.,* sidechain repacking*) to find the most favorable position for each sidechain and improve MolProbity scores^69^. Both optimizations were done with the AMOEBA polarizable force field^70,71^. We then used the optimized protein structure to calculate the protein stability change due to the amino acid changes. Folding free energy changes (i.e., *protein stability changes*) were measured using NAnoscale Molecular Dynamics (NAMD) by calculating the free energy change due to mutation for a folded and unfolded state of the wildtype and mutant protein^72^. The protein stability change (∆∆G) is defined as ∆∆G = ∆G_folded_ − ∆G_unfolded_. Generally, protein stability changes that are greater than 1 kcal/mol are more likely to cause disease. This analysis offered insight into the effect of the DNMs on protein functionality.

### Sanger-sequencing validation

To eliminate false positive DNMs, we conducted Sanger-sequencing validations of selected high impact DNMs discovered through WGS in our case-parent trios. Briefly, we designed primers around the DNMs by including 500 base pairs upstream and downstream of the mutation locus. The primers were designed using primer3 (https://primer3.ut.ee/) and optimized for the application of the regions containing the DNMs. The optimized primers were used to amplify these loci in DNA samples using a DNA concentration of 4 ng/μl in a 10 μl polymerase chain reaction. A YRI HapMap sample was added to the plate as a negative control. Details of the primers and annealing temperatures are available from the Butali laboratory upon request. The PCR products were sent to Functional Biosciences, Inc., Madison, WI (https://functionalbio.com/) for sequencing. Sequence data were investigated to confirm the DNMs.

### Gene set enrichment analysis (GSEA) and SysFACE based craniofacial gene expression analysis

We did a gene set enrichment analysis to identify those processes significantly enriched within our DNMs gene set. We used the Database for Annotation, Visualization and Integrated Discovery (DAVID) to identify the biological processes significantly enriched within gene sets with the DNMs. The list of the genes with DNMs were entered in as a query in the DAVID Bioinformatics Resources 6.8 (https://david.ncifcrf.gov/) and the analysis was run. The biological processes with at least a nominal p-value (p < 0.05) were selected, among others. Among those with suggestive significance value (p < 0.05), we identified those processes involved in lip and palatal development where genes on our list were involved.

To gain biological insights on the candidate genes among the DNM gene lists, we examined the expression in relevant craniofacial tissues using SysFACE (Systems tool for craniofacial expression-based gene discovery), as previously published^25,73^. Mouse craniofacial transcriptomics microarray data for maxilla, frontonasal and palate at embryonic (E) and post-natal (P) stages was used for examining gene expression. Transcriptomics data from public databases such as FaceBase (https://www.facebase.org) and National Center for Biotechnology Information (NCBI) Gene Expression Omnibus (GEO) (https://www-ncbi-nlm-nih-gov.udel.idm.oclc.org/geo/) was meta-analyzed as previously described^73^. The following FaceBase datasets (FB00000352, FB00000353, FB00000107, FB00000254, FB00000264, FB00000468.01, FB00000474.01, FB00000477.01, FB00000905) and NCBI Gene Expression Omnibus (GEO) datasets (GSE7759, GSE55965, GSE22989, GSE31004, GSE11400) were considered in the analysis. Craniofacial tissue expression, presented in fluorescence intensity units, were used to generate heatmap representation.

## Supplementary Information

Supplementary Information.

## Data Availability

Data available through dbGaP Accession Number: phs001997.

## Abbreviations

BAM: Binary alignment map
CADD: Combined annotation dependent depletion
CNV: Copy number variant
DAVID: Database for annotation, visualization and integrated discovery
DNM: De novo mutation
GATK: GenomeAnalysisToolKit
GMKF: Gabriella–Miller kids first
GQ: Genotype quality
GSEA: Gene set enrichment analysis
GVCF: Genome variant call format
HapMap: Haplotype map
HPE: Holoprosencephaly
HWE: Hardy–Weinberg equilibrium
Indel: Insertions or deletions
IRB: Institution Review Board
MAF: Minor allele frequency
MGI: Mouse genome informatics
NAMD: NAnoscale molecular dynamics
nsCL/P: Nonsyndromic cleft lip with or without cleft palate
nsCLO: Nonsyndromic cleft lip only
nsCLP: Nonsyndromic cleft lip and palate
nsCPO: Nonsyndromic cleft palate only
PCR: Polymerase chain reaction
PolyPhen: Polymorphism phenotyping
QC: Quality control
RD: Read depth
SAM: Sequence alignment map
SIFT: Sorting Intolerant from tolerant
SNP: Single nucleotide polymorphism
SysFACE: Systems tool for craniofacial expression
VCF: Variant call format
WGS: Whole-genome sequencing
YRI: Yoruba in Ibadan
