# Supplementary material for: Whole-genome sequencing reveals de-novo mutations associated with nonsyndromic cleft lip/palate
Source: Sci Rep. 2022 Jul 11;12:11743. doi: 10.1038/s41598-022-15885-1 (PMC9273634; doi:10.1038/s41598-022-15885-1)
Supplement: Supplementary file 1 — Supplementary Information. [file 41598_2022_15885_MOESM1_ESM.pdf]

Whole-genome Sequencing reveals *de-novo* mutations associated with Nonsyndromic Cleft Lip/Palate

Waheed Awotoye[1,2]*, Peter A. Mossey[3], Jacqueline B. Hetmanski[4], Lord J.J Gowans[5], Mekonen A. Eshete[6], Wasiu L. Adeyemo[7], Azeez Alade[2,8], Erliang Zeng[9], Olawale Adamson[7], Thirona Naicker[10], Deepti Anand[11], Chinyere Adeleke[2], Tamara Busch[2], Mary Li[2], Aline Petrin[1, 12], Babatunde S. Aregbesola[13], Ramat O. Braimah[13], Fadekemi O. Oginni[13], Ayodeji O. Oladele[13], Abimbola Oladayo[2], Sami Kayali[2], Joy Olotu[14], Mohaned Hassan[2], John Pape[2], Peter Donkor[15], Fareed K.N. Arthur[5], Solomon Obiri-Yeboah[16], Daniel K. Sabbah[17], Pius Agbenorku[15], Gyikua Plange-Rhule[18], Alexander Acheampong Oti[16], Rose A. Gogal[19], Terri H.Beaty[4], Margaret Taub[4], Mary L. Marazita[20], Michael J. Schnieders[19], Salil A. Lachke[11,21], Adebowale A. Adeyemo[22], Jeffrey C. Murray[23], Azeez Butali[1,2*]

[1]Iowa Institute for Oral Health Research, University of Iowa, Iowa City, IA, USA
[2]Department of Oral Pathology, Radiology and Medicine, College of Dentistry, University of Iowa, Iowa City, IA, USA
[3]Department of Orthodontics, University of Dundee, Dundee, UK
[4]Department of Epidemiology, Johns Hopkins Bloomberg School of Public Health, Baltimore, MD, USA
[5]Department of Biochemistry and Biotechnology, Kwame Nkrumah University of Science and Technology, Kumasi, Ghana
[6]Addis Ababa University, School Medicine, Surgical Department, Addis Ababa, Ethiopia
[7]Department of Oral and Maxillofacial Surgery, University of Lagos, Lagos Nigeria
[8]Department of Epidemiology, College of Public Health, University of Iowa, Iowa City, IA, USA.
[9]Division of Biostatistics and Computational Biology, College of Dentistry, University of Iowa, Iowa City, IA, USA
[10]Department of Pediatrics, University of KwaZulu-Natal, South Africa
[11]Department of Biological Sciences, University of Delaware
[12]Department of Orthodontics, University of Iowa, Iowa City, IA, USA
[13]Department of Oral and Maxillofacial Surgery, Obafemi Awolowo University, Ile-Ife, Osun A234, Nigeria
[14]Department of Anatomy, University of Port Harcourt
[15]Department of Surgery, School of Medicine and Dentistry, Kwame Nkrumah University of Science and Technology, Kumasi, Ghana
[16]Department of Maxillofacial Sciences, School of Medicine and Dentistry, Kwame Nkrumah University of Science and Technology, Kumasi, Ghana
[17]Department of Child Oral Health and Orthodontics, School of Medicine and Dentistry, Kwame Nkrumah University of Science and Technology, Kumasi, Ghana
[18]Department of Child Health, School of Medicine and Dentistry, Kwame Nkrumah University of Science and Technology, Kumasi, Ghana
[19]Center for Biocatalysis and Bioprocessing (CBB), University of Iowa
[20]Center for Craniofacial and Dental Genetics, Department of Oral and Craniofacial Sciences, School of Dental Medicine, and Department of Human Genetics, Graduate School of Public Health, University of Pittsburgh, Pittsburgh, PA, USA
[21]Center for Bioinformatics and Computational Biology, University of Delaware
[22]National Human Genomic Research Institute, Bethesda, MD, USA
[23]Department of Pediatrics, University of Iowa, Iowa City, IA, USA

Correspondence:

Azeez Butali, Department of Oral Pathology, Radiology and Medicine, College of Dentistry, University of Iowa, Iowa City, IA, USA Email: Azeez-butali@uiowa.edu

Chromosome Location	Ref/Alt	Gene Names	HGVS c.	HGVS p.	Effect
1:12617239	G/A	*DHRS3*	c.110C>T	p.Ser37Leu	Missense
1:22863094	C/A	*EPHB2*	c.869C>A	p.Pro290His	Missense
1:24458944	A/G	*NIPAL3*	c.830A>G	p.Tyr277Cys	Missense
1:25446845	T/G	*MACO1*	c.164T>G	p.Leu55Arg	Missense
1:52698320	C/A	*COA7*	c.7G>T	p.Gly3Cys	Missense
1:62555824	C/T	*DOCK7*	c.2596+1G>A		LoF
1:92245604	T/C	*C1orf146*	c.473T>C	p.Ile158Thr	Missense
2:108766258	A/G	*RANBP2*	c.5719A>G	p.Asn1907Asp	Missense
2:112659645	A/G	*SLC20A1*	c.1490A>G	p.Asn497Ser	Missense
2:11618333	C/T	*GREB1*	c.3458C>T	p.Pro1153Leu	Missense
2:178738241	G/A	*TTN*	c.14212C>T	p.Arg4738Ter	LoF
2:178747180	A/G	*TTN*	c.11312-5259T>C		Missense
2:200573408	C/G	*SGO2*	c.3062C>G	p.Ser1021Cys	Missense
2:21142968	T/C	*TDRD15*	c.5501T>C	p.Val1834Ala	Missense
2:214410244	G/A	*SPAG16*	c.1825G>A	p.Val609Ile	Missense
2:235883447	C/T	*AGAP1*	c.1153C>T	p.His385Tyr	Missense
2:24821592	C/T	*ADCY3*	c.3055G>A	p.Asp1019Asn	Missense
2:27581847	T/C	*C2orf16*	c.5275T>C	p.Cys1759Arg	Missense
2:47029351	A/G	*TTC7A*	c.1769A>G	p.Asn590Ser	Missense
2:88627076	C/T	*EIF2AK3*	c.199G>A	p.Ala67Thr	Missense
2:96193078	C/T	*STARD7*	c.743G>A	p.Arg248His	Missense
3:12557158	C/T	*MKRN2*	c.8C>T	p.Thr3Ile	Missense
3:129315334	G/A	*H1-10*	c.569C>T	p.Ala190Val	Missense
3:134561462	G/A	*CEP63*	c.2039G>A	p.Arg680His	Missense
3:179563094	T/G	*ACTL6A*	c.2T>G	p.Met1Arg	LoF
3:189894226	C/G	*TP63*	c.1767C>G	p.Ile589Met	Missense
3:195725943	C/T	*MUC20*	c.1340C>T	p.Thr447Met	Missense
3:195782050	A/G	*MUC4*	c.9530T>C	p.Val3177Ala	Missense
3:195786568	G/A	*MUC4*	c.5012C>T	p.Ser1671Phe	Missense
3:31990509	C/G	*ZNF860*	c.1430C>G	p.Thr477Ser	Missense
3:36830891	G/A	*TRANK1*	c.8692C>T	p.Arg2898Trp	Missense
3:50302157	G/A	*HYAL1*	c.800C>T	p.Ala267Val	Missense
3:51984131	C/T	*ACY1*	c.67C>T	p.Arg23Cys	Missense
4:121164073	G/A	*TNIP3*	c.53C>T	p.Thr18Met	Missense
4:147875102	C/T	*ARHGAP10*	c.784C>T	p.Arg262Ter	LoF
4:48382595	C/T	*SLAIN2*	c.890C>T	p.Thr297Met	Missense
4:48561507	C/T	*FRYL*	c.3826G>A	p.Ala1276Thr	Missense
4:52029703	A/G	*SGCB*	c.404T>C	p.Leu135Ser	Missense
4:5841335	C/T	*CRMP1*	c.1126G>A	p.Asp376Asn	Missense

5:13919284	G/C	*DNAH5*	c.867C>G	p.His289Gln	Missense
5:73866000	G/T	*ARHGEF28*	c.2139G>T	p.Gln713His	Missense
5:77039595	G/A	*AGGF1*	c.746G>A	p.Arg249His	Missense
6:113953409	C/G	*HDAC2*	c.507G>C	p.Gln169His	Missense
6:113953410	T/G	*HDAC2*	c.506A>C	p.Gln169Pro	Missense
6:158493629	C/T	*TULP4*	c.1688C>T	p.Ser563Phe	Missense
6:161106684	T/C	*MAP3K4*	c.4027T>C	p.Trp1343Arg	Missense
6:26107744	G/A	*H1-6*	c.350C>T	p.Pro117Leu	Missense
6:30986727	G/C	*MUC21*	c.552G>C	p.Glu184Asp	Missense
6:30986737	G/A	*MUC21*	c.562G>A	p.Val188Met	Missense
6:32530209	G/A	*HLA-DRB5*	c.16C>T	p.Leu6Phe	Missense
6:32584273	A/T	*HLA-DRB1*	c.206T>A	p.Phe69Tyr	Missense
6:54940571	C/T	*FAM83B*	c.1600C>T	p.Leu534Phe	Missense
6:98836046	A/T	*POU3F2*	c.1173A>T	p.Leu391Phe	Missense
7:121996450	G/A	*PTPRZ1*	c.997G>A	p.Glu333Lys	Missense
7:128866780	C/T	*ATP6V1FNB*	c.371C>T	p.Thr124Met	Missense
7:134947749	G/A	*CALD1*	c.1774G>A	p.Asp592Asn	Missense
7:149826185	G/A	*SSPOP*	c.14369G>A	p.Arg4790Gln	Missense
7:151023204	T/C	*ATG9B*	c.662A>G	p.Gln221Arg	Missense
7:155803204	G/A	*SHH*	c.1085C>T	p.Ser362Leu	Missense
7:78343861	C/T	*MAGI2*	c.1325G>A	p.Gly442Asp	Missense
7:80828605	T/C	*SEMA3C*	c.244A>G	p.Ile82Val	Missense
7:93134086	C/T	*SAMD9L*	c.1886G>A	p.Arg629Gln	Missense
7:97007691	G/C	*DLX6*	c.490G>C	p.Gly164Arg	Missense
8:10607950	G/A	*RP1L1*	c.6148C>T	p.Pro2050Ser	Missense
8:125083835	C/A	*WASHC5*	c.64G>T	p.Gly22Cys	Missense
8:143924216	C/T	*PLEC*	c.5794G>A	p.Glu1932Lys	Missense
8:144512000	C/A	*RECQL4*	c.3304G>T	p.Asp1102Tyr	Missense
8:144722489	T/C	*ZNF251*	c.1171A>G	p.Ser391Gly	Missense
9:121021568	C/T	*C5*	c.1243G>A	p.Asp415Asn	Missense
9:131128358	C/G	*NUP214*	c.268C>G	p.Pro90Ala	Missense
9:134887404	C/T	*FCN2*	c.931C>T	p.Arg311Ter	LoF
9:33796675	G/A	*PRSS3*	c.244G>A	p.Val82Ile	Missense
9:89381234	C/T	*SEMA4D*	c.1559G>A	p.Cys520Tyr	Missense
10:100989673	A/C	*TWNK*	c.1273A>C	p.Ile425Leu	Missense
10:110821608	G/A	*RBM20*	c.2989G>A	p.Val997Met	Missense
10:26021561	C/G	*MYO3A*	c.644C>G	p.Ser215Cys	Missense
10:5937203	C/T	*FBH1*	c.3208C>T	p.Gln1070Ter	LoF
10:69572797	T/C	*NEUROG3*	c.247A>G	p.Ser83Gly	Missense
10:71730574	A/T	*CDH23*	c.3685A>T	p.Ile1229Phe	Missense
10:90915813	G/A	*ANKRD1*	c.719C>T	p.Ala240Val	Missense

10:97057258	G/A	*SLIT1*	c.1109C>T	p.Thr370Ile	Missense
11:1188914	G/A	*MUC5AC*	c.10769G>A	p.Arg3590His	Missense
11:55344004	A/C	*OR4A16*	c.804A>C	p.Leu268Phe	Missense
11:65389453	G/A	*FRMD8*	c.178G>A	p.Ala60Thr	Missense
11:6567652	G/A	*DNHD1*	c.12143G>A	p.Arg4048Gln	Missense
11:66715280	A/T	*SPTBN2*	c.425T>A	p.Val142Glu	Missense
11:84534687	T/A	*DLG2*	c.402A>T	p.Leu134Phe	Missense
11:95979264	T/C	*MAML2*	c.3155A>G	p.Asn1052Ser	Missense
12:110614214	T/C	*TCTN1*	c.32T>C	p.Val11Ala	Missense
12:47796247	T/C	*HDAC7*	c.755A>G	p.Asn252Ser	Missense
12:49050509	G/A	*KMT2D*	c.3079C>T	p.Leu1027Phe	Missense
12:50352111	G/T	*FAM186A*	c.4721C>A	p.Ala1574Asp	Missense
12:50352111	G/T	*FAM186A*	c.4721C>A	p.Ala1574Asp	Missense
12:50352857	G/C	*FAM186A*	c.3975C>G	p.Ile1325Met	Missense
12:63802407	T/A	*RXYLT1*	c.743+2T>A		LoF
13:102875878	C/T	*ERCC5*	c.3536C>T	p.Ala1179Val	Missense
13:19835686	G/A	*ZMYM5*	c.1042C>T	p.Arg348Cys	Missense
13:95763714	G/A	*DNAJC3*	c.920G>A	p.Arg307His	Missense
14:104948630	G/A	*AHNAK2*	c.6821C>T	p.Ala2274Val	Missense
14:104948633	G/C	*AHNAK2*	c.6818C>G	p.Thr2273Arg	Missense
14:104948637	C/T	*AHNAK2*	c.6814G>A	p.Val2272Met	Missense
14:23990277	C/T	*DHRS4L2*	c.224C>T	p.Thr75Met	Missense
14:53093367	T/C	*DDHD1*	c.1111A>G	p.Thr371Ala	Missense
14:57208791	T/C	*EXOC5*	c.1945A>G	p.Met649Val	Missense
14:78967390	A/T	*NRXN3*	c.2960A>T	p.Asp987Val	Missense
14:81144181	G/C	*TSHR*	c.2123G>C	p.Gly708Ala	Missense
15:40200274	A/T	*BUB1B*	c.1432A>T	p.Thr478Ser	Missense
15:58821727	T/C	*MINDY2*	c.1133T>C	p.Ile378Thr	Missense
15:78180855	G/A	*ACSBG1*	c.1153C>T	p.Arg385Cys	Missense
15:88856906	A/G	*ACAN*	c.4321A>G	p.Thr1441Ala	Missense
15:89582731	C/G	*TICRR*	c.700C>G	p.Leu234Val	Missense
16:1775841	G/A	*EME2*	c.824G>A	p.Arg275His	Missense
16:2255717	T/C	*RNPS1*	c.686A>G	p.Asp229Gly	Missense
16:28538847	C/A	*NUPR1*	c.61G>T	p.Asp21Tyr	Missense
16:48138240	T/G	*ABCC12*	c.967A>C	p.Asn323His	Missense
16:5056123	C/G	*C16orf89*	c.693G>C	p.Met231Ile	Missense
16:53156715	A/G	*CHD9*	c.626A>G	p.Asn209Ser	Missense
16:67645262	G/A	*CARMIL2*	c.16G>A	p.Asp6Asn	Missense
16:9763618	C/T	*GRIN2A*	c.3926G>A	p.Arg1309Gln	Missense
17:19019527	C/T	*SLC5A10*	c.1394C>T	p.Ser465Phe	Missense
17:3216519	G/A	*OR1A1*	c.899G>A	p.Arg300Gln	Missense

17:33291652	C/T	*ASIC2*	c.464G>A	p.Gly155Asp	Missense
17:4032234	C/T	*ZZEF1*	c.6784G>A	p.Val2262Ile	Missense
17:41756183	T/C	*JUP*	c.2078A>G	p.Tyr693Cys	Missense
17:41821726	C/A	*FKBP10*	c.1472C>A	p.Thr491Lys	Missense
17:41912433	C/T	*ACLY*	c.269G>A	p.Gly90Glu	Missense
17:4884399	G/T	*MINK1*	c.343G>T	p.Asp115Tyr	Missense
17:4887701	C/T	*MINK1*	c.1141C>T	p.Arg381Ter	LoF
17:68921472	C/T	*ABCA8*	c.1522G>A	p.Glu508Lys	Missense
17:7284563	G/A	*SLC2A4*	c.806G>A	p.Arg269His	Missense
17:75809893	G/A	*UNK*	c.238G>A	p.Gly80Ser	Missense
17:9802513	A/G	*GSG1L2*	c.755T>C	p.Phe252Ser	Missense
18:35677701	G/A	*GALNT1*	c.425G>A	p.Arg142His	Missense
18:57701307	G/A	*ATP8B1*	c.400C>T	p.Pro134Ser	Missense
18:62211239	G/A	*RELCH*	c.613G>A	p.Ala205Thr	Missense
18:62570232	G/T	*ZCCHC2*	c.1975+1G>T		LoF
18:63793232	C/A	*SERPINB7*	c.291C>A	p.Ser97Arg	Missense
19:10403376	A/G	*CDC37*	c.102+2T>C		LoF
19:10514953	T/C	*S1PR5*	c.59A>G	p.Asn20Ser	Missense
19:12792168	G/A	*JUNB*	c.397G>A	p.Glu133Lys	Missense
19:13983583	C/T	*RFX1*	c.332G>A	p.Arg111Gln	Missense
19:20807211	C/A	*ZNF66*	c.1611C>A	p.His537Gln	Missense
19:20807211	C/A	*ZNF66*	c.1611C>A	p.His537Gln	Missense
19:20807213	A/T	*ZNF66*	c.1613A>T	p.Lys538Met	Missense
19:20807213	A/T	*ZNF66*	c.1613A>T	p.Lys538Met	Missense
19:35785048	C/T	*ARHGAP33*	c.1663C>T	p.Arg555Trp	Missense
19:41197899	G/A	*CYP2S1*	c.464G>A	p.Cys155Tyr	Missense
19:54575487	A/C	*LILRA2*	c.887A>C	p.Tyr296Ser	Missense
19:54773524	A/G	*KIR2DL1*	c.262A>G	p.Ser88Gly	Missense
19:54775225	A/G	*KIR2DL1*	c.431A>G	p.Asn144Ser	Missense
19:57874619	C/G	*ZNF814*	c.771G>C	p.Leu257Phe	Missense
19:58516973	G/A	*ZBTB45*	c.701C>T	p.Pro234Leu	Missense
19:8499546	C/A	*PRAM1*	c.262G>T	p.Val88Phe	Missense
19:8499546	C/A	*PRAM1*	c.262G>T	p.Val88Phe	Missense
19:8499546	C/A	*PRAM1*	c.262G>T	p.Val88Phe	Missense
19:8951340	G/C	*MUC16*	c.25430C>G	p.Ala8477Gly	Missense
20:46013314	C/A	*MMP9*	c.1390C>A	p.Pro464Thr	Missense
20:58854572	C/A	*GNAS*	c.1307C>A	p.Ala436Asp	Missense
21:44943819	G/A	*FAM207A*	c.265G>A	p.Val89Ile	Missense
22:29489649	T/A	*NEFH*	c.2009T>A	p.Val670Glu	Missense
22:38645588	C/T	*FAM227A*	c.200G>A	p.Arg67His	Missense
22:45328021	G/C	*FAM118A*	c.480G>C	p.Gln160His	Missense

Supplementary Table 1: List of *de novo* mutations (DNMs) identified in nsCL/P African case-parent trios through analysis of the Whole-genome sequence.

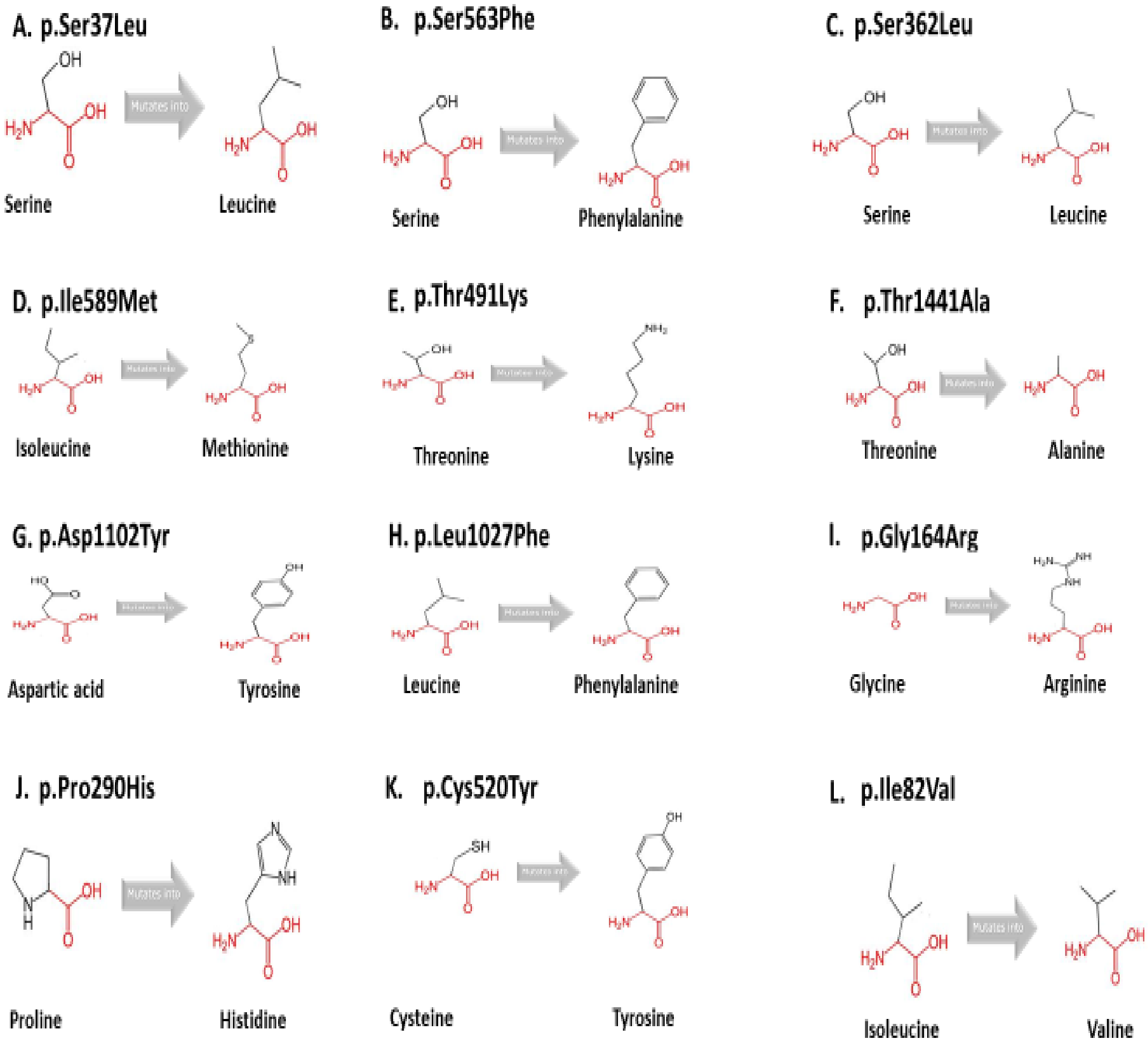

Supplementary Figure 1: Predicted effects of the DNMs on protein structures and functions. A: Deleterious p.Ser37Leu DNM in *DHRS3* found in the catalytic domain. B: Damaging p.Ser563Phe DNM in *TULP4* disrupts the secondary structure of the polypeptide chain. C: Deleterious p.Ser362Leu DNM in *SHH* disrupts the secondary structure of the protein. D: Deleterious p.Ile589Met DNM in *TP63* found in the sterile alpha motif (SAM) domain. This mutation cause a disruption in the protein interaction of this domain. E: p.Thr491Lys

DNM in *FKBP10* which cause a disruption in the structure of the protein. **F**: p.Thr1441Ala DNM in *ACAN* which likely affect the protein structure and function. **G**: Damaging p.Asp1102Tyr DNM in *RECQL4* which affect the charge thus affecting the interactive function of the protein. **H**: Deleterious p.Leu1027Phe DNM in *KMT2D* affects the structure and interaction with other proteins. **I**: Deleterious p.Gly164Arg DNM in *DLX6* affects the protein interaction function of the Homeobox-like domain. **J**: Damaging p.Pro290His DNM in the *EPHB2* alter the special conformation of the protein and interaction. **K**: Damaging p.Cys520Tyr DNM in the *SEMA4D* found in the highly conserved PSI domain. The wildtype amino acid residue is highly conserved at this location and mutation could cause a disruption in the binding function of the PSI domain. **L**: p.Ile82Val DNM in *SEMA3C* is found within the protein-binding domain thus it may abolish the function.

A

	E10.5 Frontonasal	E11.0 Frontonasal	E11.5 Frontonasal	E10.0 Mandible	E10.5 Mandible	E10.5 Mandible (lateral)	E10.5 Mandible (medial)	E11.0 Mandible	E11.5a Mandible	E11.5b Mandible
Actl6a	2059.0	2286.8	1976.1	3464.7	1918.2	2578.5	3039.4	2330.2	3285.8	1768.0
Ankrd1	49.2	38.9	36.1	38.5	50.9	32.4	32.3	34.9	41.5	40.3
Bivm	590.2	771.6	567.6	873.5	535.1	915.8	762.4	695.2	1064.1	540.8
Bub1b	2332.1	2807.7	2402.0	3662.5	2129.0	2684.9	2569.4	2656.8	1878.6	2085.3
Cdc37	4071.1	4213.5	3833.5	5563.0	3921.9	4100.8	3897.7	4387.9	2701.7	3694.0
Cdh23	89.7	74.9	77.8	63.6	75.9	92.0	66.0	81.3	66.6	74.8
Cep63	457.0	576.5	464.1	742.9	394.4	446.5	467.1	467.6	748.0	387.4
Coa7	410.0	394.4	293.3	744.9	382.9	507.7	540.9	397.9	676.7	328.5
Ddhd1	103.3	144.4	125.2	91.8	145.8	95.4	80.2	109.4	75.2	122.8
Dnah5	65.0	51.5	55.5	42.8	62.8	42.3	41.5	56.7	46.9	64.1
Dnajc3	381.5	434.7	403.5	767.6	365.7	863.4	908.2	489.1	1258.5	369.0
Dnhd1	93.0	83.1	82.3	68.0	98.9	86.1	71.2	91.8	70.7	88.9
Dock7	1043.0	1237.2	1319.7	1533.6	1058.0	1980.8	2377.4	1334.0	2055.9	1381.5
Ercc5	249.8	350.1	327.4	452.8	236.6	374.8	405.8	339.1	544.3	301.7
Exoc5	933.0	1096.2	1108.5	1401.7	839.2	1126.6	1349.5	1066.3	1739.1	1001.2
Fcn2 (Fcnb)	66.8	60.8	58.5	60.7	66.3	76.2	51.3	65.9	54.6	63.1
Gnas	6157.7	8365.7	9898.6	11273.9	6402.0	10760.8	13403.9	10819.1	10843.6	9792.3
Junb	49.9	43.7	41.7	84.8	39.6	77.4	72.3	47.1	56.4	39.9
Jup	583.5	472.6	639.2	363.9	573.1	269.8	284.2	487.3	265.8	543.4
Map3k4	284.2	460.6	399.6	719.0	237.3	1384.5	1284.9	479.8	1425.2	333.3
Mkrn2	571.9	680.0	651.9	759.1	525.0	737.7	722.8	654.9	842.0	621.2
Mmp9	77.5	73.0	79.3	56.9	86.2	62.6	71.6	76.0	88.4	89.8
Muc4	39.1	31.7	58.4	21.7	44.9	18.1	20.3	25.2	22.9	46.6
Nefh	45.0	31.7	30.5	18.2	41.9	17.9	17.1	26.0	16.8	35.3
Neurog3	61.7	57.6	51.6	35.6	80.6	32.2	27.8	54.7	34.1	55.6
Nipal3	125.5	141.1	145.6	154.5	98.1	163.3	182.5	122.6	152.1	119.3
Ptprz1	84.7	93.8	108.7	166.7	94.6	156.3	284.4	112.4	263.7	110.0
Serpinb7	25.1	29.3	23.1	19.4	30.3	16.6	17.6	22.6	16.5	24.2
Slc20a1	963.4	1214.2	1102.1	1547.6	821.2	1481.7	1795.4	1202.1	1109.5	1032.6
Tctn1	142.5	156.6	167.0	218.9	130.7	232.7	286.0	157.0	113.8	164.6
Zcchc2	105.4	159.0	159.6	245.4	93.5	397.8	314.9	147.2	459.3	127.2

	E11.5 Mandible (medial)	E10.5 Maxilla	E11.0 Maxilla	E11.5 Maxilla	E13.5a Palate	E13.5b Palate	E14.5a Palate	E14.5b Palate	E14.5c Palate	P0 Palate
Actl6a	2621.8	1820.3	2212.0	2329.0	2096.7	1941.4	1281.1	1581.7	1419.8	956.3
Ankrd1	48.3	42.6	42.1	35.6	52.3	37.7	43.5	39.6	40.4	468.6
Bivm	607.2	513.5	702.2	686.5	946.2	944.2	456.0	611.5	757.9	501.8
Bub1b	2361.7	2191.4	2583.7	2589.7	1133.4	1007.8	1176.3	1175.6	1426.6	552.4
Cdc37	3921.6	3717.3	4796.3	4432.8	2305.8	1609.8	3131.6	3572.0	3776.8	1873.4
Cdh23	80.0	80.4	71.7	74.0	56.2	61.1	59.9	54.8	56.3	69.2
Cep63	436.7	430.6	538.6	503.8	688.5	690.6	437.0	458.1	437.3	546.6
Coa7	602.9	340.5	407.9	332.4	520.6	444.3	252.3	315.3	372.5	431.0
Ddhd1	119.7	132.4	111.1	116.3	70.4	98.5	125.4	201.2	88.0	117.1
Dnah5	37.2	60.2	57.1	53.3	36.8	37.0	44.7	35.5	35.0	30.8
Dnajc3	778.0	394.5	435.7	388.8	1641.7	1400.1	1387.2	1278.3	1198.2	1789.9
Dnhd1	69.4	96.0	82.4	77.6	82.9	69.1	93.2	94.0	85.9	61.7
Dock7	2210.4	1033.2	1115.4	1381.2	1599.8	2198.2	2154.8	2180.2	2247.0	989.9
Ercc5	403.9	229.7	391.8	444.8	891.3	909.3	455.9	544.4	438.4	301.3
Exoc5	1276.5	862.4	1089.3	1233.7	1618.5	1755.6	1355.8	1444.1	1130.0	1436.2
Fcn2 (Fcnb)	52.0	64.6	58.2	58.8	60.4	57.1	56.7	48.9	57.4	174.5
Gnas	13207.7	6086.8	7397.4	9648.2	11017.9	10341.4	13377.6	13094.1	11680.8	11379.1
Junb	75.0	40.3	45.2	44.5	61.3	58.6	59.7	72.9	87.0	141.7
Jup	273.4	558.1	536.9	545.8	330.2	280.0	363.5	527.2	586.5	536.0
Map3k4	1268.9	309.8	447.1	456.0	1405.1	1428.4	891.5	1047.3	1288.7	1006.9
Mkrn2	815.5	514.6	666.9	718.4	892.6	686.5	675.5	632.2	764.3	712.4
Mmp9	78.6	91.5	77.5	73.3	407.7	207.0	439.7	320.4	751.5	325.1
Muc4	27.1	48.2	32.0	47.6	25.1	25.5	65.6	141.0	26.7	23.8
Nefh	18.5	42.0	33.7	26.6	15.7	15.7	18.2	22.6	52.0	136.3
Neurog3	33.2	74.1	58.8	41.6	27.1	31.1	33.3	24.8	30.7	29.0
Nipal3	144.5	115.9	118.6	125.3	188.6	140.7	154.4	152.1	200.5	322.6
Ptprz1	64.5	83.4	81.1	106.0	655.9	690.9	728.9	685.4	890.3	301.3
Serpinb7	17.1	27.0	25.5	23.6	53.6	19.4	42.8	54.7	90.2	32.6
Slc20a1	1373.6	963.7	1192.8	1207.6	883.1	780.6	1115.8	1260.7	891.9	1088.1
Tctn1	247.1	137.4	146.0	150.7	182.4	177.3	506.5	307.7	362.1	168.8
Zcchc2	341.3	103.4	125.4	154.4	581.2	559.8	387.2	443.9	343.8	425.9

B

	E10.5 Mandibular Columnar Epithelium	E10.5 Maxillary Columnar Epithelium
Actl6a	2804.5	2204.1
Ankrd1	37.8	36.4
Bivm	140.4	29.4
Bub1b	219.6	186.2
Cdc37	2574.8	2210.9
Cdh23	50.8	55.2
Cep63	240.4	321.5
Coa7	1301.6	1162.9
Ddhd1	45.3	41.1
Dnah5	39.7	45.6
Dnajc3	382.3	150.8
Dock7	72.6	73.1
Ercc5	146.5	137.3
Fcn2 (Fcnb)	83.3	86.0
Gnas	518.0	588.7
Junb	115.7	192.2
Jup	174.7	197.2
Map3k4	79.6	89.3
Mkrn2	442.2	547.8
Mmp9	62.6	74.4
Muc4	58.2	63.7
Nefh	61.3	77.4
Neurog3	32.8	45.6
Nipal3	39.0	47.0
Ptprz1	34.5	29.9
Serpinb7	15.7	15.8
Slc20a1	107.2	112.8
Slit1	57.3	75.6
Tctn1	107.9	111.5
Zcchc2	30.1	37.0

C

	E10.5 Lateral Nasal Eminence	E10.5 Medial Nasal Prominence	E10.5 Mandibular Arch	E10.5 Mandibular Arch Epidermal Ectoderm	E10.5 Maxillary Arch	E10.5 Maxillary Arch Epidermal Ectoderm
Actl6a	923.3	302.9	215.7	306.1	365.5	119.9
Ankrd1	473.4	638.4	566.9	254.2	368.3	317.0
Bivm	173.5	157.4	168.0	122.0	196.7	183.2
Bub1b	43.4	37.3	32.4	39.7	37.5	51.8
Cdc37	23.1	23.8	20.6	27.2	21.0	31.1
Cdh23	21.0	19.9	20.7	22.7	20.0	23.2
Cep63	20.5	18.3	19.9	19.5	22.1	21.2
Coa7	38.6	37.5	39.1	38.5	38.4	41.5
Ddhd1	605.3	579.4	750.1	592.3	752.2	695.2
Dnah5	840.0	1070.8	680.5	956.7	666.7	891.9
Dnajc3	3131.0	3131.4	3178.6	3966.5	1833.6	3860.8
Dock7	363.2	29.3	98.2	32.3	28.6	29.7
Ercc5	202.1	251.5	257.3	171.8	214.1	167.2
Exoc5	176.8	156.0	161.5	159.4	198.9	188.3
Fcn2 (Fcnb)	81.0	70.5	72.4	58.9	82.9	82.5
Gnas	26.6	25.9	22.4	25.8	25.4	27.7
Gsg1l2	40.7	33.6	34.6	39.9	39.5	37.2
Junb	78.9	60.6	73.3	66.1	89.6	89.3
Jup	90.6	90.5	91.3	72.9	103.0	105.5
Map3k4	57.0	59.2	52.6	88.6	67.2	59.5
Mkrn2	93.6	65.3	71.1	70.1	118.1	78.7
Mmp9	19.0	16.2	16.4	18.5	16.8	20.9
Muc4	90.6	92.6	91.4	78.3	95.0	99.9
Nefh	45.9	42.4	43.2	40.6	52.7	48.0
Neurog3	24.9	24.5	21.6	25.0	19.6	31.7
Nipal3	24.7	21.4	20.9	26.6	21.9	25.2
Ptprz1	143.3	457.1	193.6	230.0	118.4	572.5
Serpinb7	40.8	39.0	39.4	38.0	48.1	45.3
Slc20a1	126.5	92.9	102.4	99.0	119.3	142.1
Slit1	42.6	39.3	39.3	50.9	43.7	51.4
Tctn1	23.5	21.3	23.4	20.6	21.8	24.3
Zcchc2	123.1	140.7	200.0	145.5	142.6	55.9

Supplementary Figure 2: SysFACE-based expression analysis of candidate genes in mouse facial

generated on the Affymetrix Mouse Genome 430 2.0 Array platform, (**B**) FaceBase microarray data generated on the Affymetrix Mouse Gene 1.0 ST Array platform, and (**C**) GSE55965 microarray data generated on the Affymetrix Mouse Gene 1.0 ST Array platform. Heat-map denotes row-wise comparative expression of individual genes in different tissues at Embryonic (E) and/or postnatal (P) stages. Intensity of the color in the heat-map is representative of candidate gene expression and the average fluorescence signal intensity is shown.
